# KIF1B Regulates NLRP3‐Mediated Pyroptosis in Asthma Progression

**DOI:** 10.1111/jcmm.70975

**Published:** 2025-12-12

**Authors:** Junchao Wang, Yuan Gao, Jing Li, Chiqiu Jiang

**Affiliations:** ^1^ Department of Pediatrics, the Third People's Hospital of Hubei Province Hubei Province China

**Keywords:** airway inflammation, asthma, KIF1B, NLRP3 inflammasome, pyroptosis

## Abstract

Asthma is a chronic inflammatory respiratory disorder triggered by allergens or environmental pollutants, characterised by airway obstruction, increased airway resistance and breathing difficulties. Although substantial progress has been made in elucidating its pathophysiology, the molecular mechanisms underlying asthma progression remain incompletely understood, and no curative therapies are currently available. The present study explored the role of KIF1B (kinesin family member 1B) in asthma pathogenesis using integrated approaches involving human cohort datasets, in vitro airway epithelial cell models and an in vivo ovalbumin (OVA)‐induced asthma mouse model. KIF1B knockdown and NLRP3 (NLR family pyrin domain‐containing 3) overexpression assays were performed to delineate the molecular mechanisms by which KIF1B modulates pyroptosis. Results showed that KIF1B expression was markedly elevated in bronchial biopsies from asthma patients, OVA‐challenged mouse lungs and IL‐13–stimulated BEAS‐2B cells. Silencing KIF1B significantly attenuated OVA‐ and IL‐13–induced oxidative stress, proinflammatory cytokine release and pulmonary injury. Specifically, KIF1B knockdown reduced the expression of pyroptosis‐associated proteins—NLRP3, cleaved caspase‐1 and cleaved gasdermin D (GSDMD)—while decreasing TNF‐α, IL‐1β and IL‐18 levels and restoring the anti‐inflammatory cytokine IL‐10. Mechanistically, NLRP3 overexpression abolished the anti‐inflammatory and cytoprotective effects of KIF1B silencing, confirming that KIF1B promotes asthmatic inflammation through activation of the NLRP3 inflammasome. In conclusion, these findings identify KIF1B as a key regulator of airway inflammation and pyroptosis in asthma via NLRP3‐dependent signalling. Targeting KIF1B may therefore represent a promising therapeutic strategy for controlling asthma progression.

## 1 Introduction

Asthma is a chronic inflammatory respiratory disorder triggered by allergens or environmental pollutants, characterised by airway obstruction, increased airway resistance and breathing difficulties [1]. The disease pathogenesis involves complex interactions between genetic susceptibility, environmental triggers and immune system dysfunction, resulting in airway hyperresponsiveness, excessive mucus production and subsequent airway obstruction and increased resistance that ultimately compromise respiratory function [2]. Common environmental triggers include allergens, air pollutants, respiratory infections and occupational irritants, all of which can precipitate acute exacerbations in susceptible individuals [3]. Current therapeutic approaches primarily focus on symptom control and exacerbation prevention through the use of anti‐inflammatory medications, bronchodilators and trigger avoidance strategies [4]. However, despite the availability of these evidence‐based interventions, a significant proportion of patients continue to experience persistent symptoms and reduced quality of life [5], highlighting the urgent need for novel therapeutic targets and more effective treatment modalities.

Despite significant advances in understanding the pathophysiology of asthma, the molecular mechanisms driving these pathological responses remain incompletely understood. It has been well established that asthma results from chronic inflammation, with aberrant T helper type 2 (Th2) inflammation representing one of the most critical mechanisms underlying the asthmatic process [6, 7]. Approximately 50% of moderate asthma cases are associated with Th2 inflammation, characterised by increased cytokine levels including IL‐4, IL‐5 and IL‐13, as well as increased IgE production [6, 8, 9, 10]. Additionally, NLRP3 (NLR family pyrin domain containing 3) inflammasome activation has been shown to drive severe asthma through the regulation of IL‐1β and IL‐18 cytokine levels [11, 12, 13]. Interestingly, emerging evidence has demonstrated that asthma pathophysiology is intimately linked to the biological circadian clock, which regulates inflammatory responses in a time‐dependent manner [11, 12, 13]. Notably, one of the key clock genes, Rev‐erbα, has been shown to modulate allergic asthmatic progression by regulating airway inflammation and mucus production in vivo [14], while also controlling the tight junction integrity of bronchial epithelium in vitro [15]. Continued investigation of molecular pathways involved in asthmatic progression is essential to reveal novel signalling mechanisms and identify additional potential therapeutic targets.

KIF1B (kinesin family member 1B) is a microtubule‐based motor protein primarily involved in the intracellular transport of proteins and organelles, particularly within neuronal cells [16]. However, its implication in respiratory diseases and inflammatory pathways has not been investigated. Our initial analysis of human asthma cohort datasets indicated significant upregulation of KIF1B in bronchial biopsies from asthmatic patients compared to healthy controls, suggesting a potential role in asthma pathogenesis. Therefore, the objective of this study was to investigate the specific role of KIF1B in asthma progression and determine whether it regulates inflammation and pyroptosis in asthmatic responses. We employed KIF1B silencing strategies in both in vitro and in vivo models to assess its functional significance in asthmatic inflammation. Additionally, NLRP3 overexpression experiments were conducted to elucidate the molecular mechanism by which KIF1B mediates inflammatory pathways in asthma pathogenesis. Our findings demonstrate that KIF1B plays a crucial role in promoting asthmatic inflammation through NLRP3‐mediated pyroptosis, identifying KIF1B as a novel therapeutic target for asthma intervention.

## 2 Methods

### 2.1 The Asthma Cohort Dataset Analysis

GSE147878 [17], based on the GPL570 platform (Affymetrix Human Genome U133 Plus 2.0 Array), was retrieved from the Gene Expression Omnibus (GEO) database. The dataset comprised bronchial biopsy samples from 60 asthmatic patients and 13 healthy controls. The raw CEL files were normalised using the Robust Multichip Average (RMA) algorithm, and differential gene expression was calculated using appropriate statistical methods and visualised using R (v 4.1.1). Log2‐transformed expression values were used for analysis, and statistical significance was determined using appropriate parametric or nonparametric tests as indicated. Gene expression differences between asthmatic patients and healthy controls were considered statistically significant at *p* < 0.05. The human dataset (GSE147878) used in this study is publicly available in the Gene Expression Omnibus (GEO) and does not require additional ethical approval as per institutional guidelines.

### 2.2 Cell Culture, Transfection and Treatment

BEAS‐2B (normal human bronchial epithelium) cells were purchased from ATCC and maintained in DMEM medium supplemented with 10% FBS and 1% penicillin–streptomycin at 37°C under normoxic conditions (21% O2) with 5% CO2 and 95% humidity. Cells were routinely tested for mycoplasma contamination using PCR‐based detection methods, and only mycoplasma‐free cultures were used for experiments. Cells between passages 3 and 8 were used for all experiments to ensure consistency. Prior to transfection and treatment, cells were seeded at 1.5 × 10^5 cells per well in 6‐well plates and allowed to adhere overnight. Upon reaching approximately 60%–70% confluency, cells were transfected with si‐KIF1B (1:1000 dilution, Cat# TL303695, OriGene, using si‐NC as the control) or NLRP3 ORF‐clone (1:1000 dilution, Cat# RC223212, OriGene, using empty vector as the control) using Lipofectamine 3000 reagent according to the manufacturer's protocol. Following transfection (48 h), when cells reached approximately 90% confluency, the medium was replaced with DMEM containing 1% FBS for serum starvation overnight (16–18 h) to synchronise cellular responses. Subsequently, cells were treated with recombinant human IL‐13 at 10 ng/mL for 24 h. Following treatment, conditioned medium was collected for cytokine measurement via ELISA, while cells were harvested and lysed for analysis of MDA, GSH, protein and gene expressions. For intracellular ROS measurement, cells were trypsinised and processed immediately for flow cytometric analysis.

### 2.3 Cell Viability Assay

BEAS‐2B cells were seeded in 96‐well plates at a density of 5 × 10^3^ cells per well and incubated overnight at 37°C with 5% CO₂. Cells were then treated with serial two‐fold dilutions of IL‐13 ranging from 0.78125 to 400 ng/mL for 24 h. Cell viability was assessed using the CCK‐8 assay kit (Cat. No. CK04, Dojindo, Japan) according to the manufacturer's instructions. Briefly, 10 μL of CCK‐8 solution was added to each well and incubated for 2 h at 37°C. Absorbance was measured at 450 nm using a microplate reader (BioTek, USA). Cell viability was calculated as a percentage relative to untreated control cells, and the IC50 value was determined using GraphPad Prism software. Each experiment was performed in triplicate and repeated three times independently.

### 2.4 RNA Extraction and Quantitative Real‐Time PCR (qRT‐PCR)

Lung tissue (approximately 30–50 mg) or cells (1 × 10^6 cells per sample) were lysed in QIAzol reagent (Cat# 79306, Qiagen) for 30 min and subsequently mixed with chloroform for 15 s, followed by centrifugation at 15,000 g for 15 min at 4°C. The aqueous phase was collected and mixed thoroughly with an equal volume of isopropanol for precipitation at −20°C for 1 h. The mixture was then centrifuged at 15,000 g for 15 min at 4°C, and the resulting RNA pellet was washed once with 75% ethanol. Subsequently, RNA pellets were resuspended in 50 μL of RNase‐free water. RNA concentrations and purity were determined using a NanoDrop spectrophotometer (ND‐1000, NanoDrop Technologies). RNA samples with A260/A280 ratios between 1.8 and 2.2 were considered acceptable for downstream applications. A total of 500 ng of RNA was used for each sample to perform qRT‐PCR. RNA was reverse transcribed using TaqMan Reverse Transcription Assay (Cat# N8080234, ThermoFisher) according to the manufacturer's protocol. Quantitative real‐time PCR was performed using TaqMan Fast Advanced Master Mix for qPCR (Cat# 4444557, ThermoFisher) on a real‐time PCR system. Gene‐specific TaqMan probes for KIF1B (Assay ID: Hs01114511_m1 for human, Mm00801827_m1 for mouse, ThermoFisher) were used, with GAPDH (Assay ID: Hs02786624_g1 for human, Mm99999915_g1 for mouse, ThermoFisher) serving as the housekeeping gene control. The reverse transcription protocol consisted of incubation at 16°C for 30 min, followed by 42°C for 30 min and reaction termination at 85°C for 5 min. For qPCR amplification, cDNA samples were subjected to an initial incubation at 50°C for 2 min and 95°C for 10 min, followed by 40 cycles of 95°C for 10 s and 60°C for 1 min. Relative gene expression levels were calculated using the comparative Ct method (2^‐ΔΔCt).

### 2.5 Protein Isolation and Immunoblotting

Snap‐frozen lung tissue (approximately 50–100 mg) or cells (2 × 10^6 cells per sample) were lysed in RIPA buffer supplemented with protease and phosphatase inhibitor cocktails (1:100 dilution). Tissue samples were homogenised using a tissue homogeniser, while cell pellets were lysed by vortexing and incubation on ice for 30 min. Lysates were centrifuged at 12,000 g for 15 min at 4°C to remove debris. Protein concentrations in the supernatants were determined using a BCA Protein Assay Kit (Cat# 23227, ThermoFisher Scientific). Equal amounts of protein (20 μg per lane) were loaded and separated by 8%–12% sodium dodecyl sulphate‐polyacrylamide gel electrophoresis (SDS‐PAGE) under reducing conditions, then transferred to 0.45 μm nitrocellulose membranes using a wet transfer system. Following transfer, membranes were blocked with 5% BSA in TBS‐T for 1 h at room temperature. Subsequently, membranes were incubated with primary antibodies: anti‐KIF1B (1:1000, ab234869, Abcam), anti‐NLRP3 (1:1000, ab263899, Abcam), anti‐cleaved caspase‐1 (1:1000, #4199, Cell Signaling Technologies) and anti‐GSDMD‐N (1:1000, #36425, Cell Signaling Technologies) overnight at 4°C with gentle agitation. The following day, membranes were washed with TBS‐T for 4 cycles of 10 min each, and subsequently incubated with horseradish peroxidase‐conjugated secondary antibody (goat anti‐rabbit, 1:10,000, #1706515, Bio‐Rad) for 1 h at room temperature. Protein bands were visualised using Pierce ECL Western Blotting Substrate and detected using a Bio‐Rad ChemiDoc MP imaging system. β‐actin (1:1000, ab8277, Abcam) served as the loading control for normalisation. Band intensities were quantified using ImageJ software and expressed as fold changes relative to the control group.

### 2.6 Reactive Oxygen Species (ROS) Measurement

The level of ROS was measured using the 2′,7′‐dichlorofluorescin diacetate (H2DCF‐DA) kit purchased from Abcam (ab113851) according to the manufacturer's protocol. Briefly, cells (1 × 10^5 cells per sample) and tissue lysates (50 mg tissue equivalent) were incubated with H2DCF‐DA reagent for 45 min at 37°C in the dark. Subsequently, fluorescent intensity was measured at excitation/emission wavelengths of 485/535 nm using a microplate reader. ROS levels were normalised to total protein content and expressed as fold changes relative to control groups.

### 2.7 Malondialdehyde (MDA) Measurement

The MDA assay kit (Cat# ab118970, Abcam) was used according to the manufacturer's protocol to measure MDA levels as an indicator of lipid peroxidation. Cell lysates (1 × 10^5 cells per sample) or tissue homogenates (50 mg tissue equivalent) were prepared in MDA Lysis Buffer and homogenised on ice. Samples were then mixed with thiobarbituric acid (TBA) solution and incubated at 95°C for 60 min to form MDA‐TBA adducts. After cooling to room temperature, the samples were centrifuged, and the supernatant was collected. The MDA‐TBA adduct levels were measured at an optical density of 532 nm. MDA concentrations were calculated using a standard curve, normalised to total protein content and expressed as nmol/mg protein.

### 2.8 Superoxide Dismutase (SOD) Measurement

The SOD Activity Assay Kit (Colorimetric, Cat# ab65354, Abcam) was used to measure SOD activity based on the manufacturer's protocol. Cell lysates (1 × 10^5 cells per sample) or tissue homogenates (50 mg tissue equivalent) were prepared in cold 20 mM HEPES buffer (pH 7.2) containing 1 mM EGTA, 210 mM mannitol, and 70 mM sucrose. Following treatment, samples were centrifuged at 1500 g for 5 min at 4°C to remove debris. The assay was performed by adding 20 μL of sample to each well, followed by 160 μL of diluted Radical Detector solution and 20 μL of diluted Xanthine Oxidase solution. The reaction mixture was incubated at 37°C for 20 min, and absorbance was measured at an optical density of 450 nm using a microplate reader. SOD activity was calculated based on the inhibition of superoxide radical formation and normalised to total protein content, expressed as U/mg protein.

### 2.9 Glutathione (GSH) Measurement

GSH levels were determined using a total glutathione assay kit purchased from Abcam (Cat# ab138881). Cell lysates (1 × 10^5 cells per sample) or lung tissue homogenates (50 mg tissue equivalent) were prepared in GSH assay buffer and deproteinised using the provided deproteinisation sample preparation kit to remove interfering proteins. The reaction mixture was prepared by combining GSH assay buffer, GSH substrate and glutathione reductase enzyme. Samples and GSH standards were added to the reaction mixture and incubated at room temperature for 10 min to allow the enzymatic reaction to proceed. Absorbance was measured at 405 nm using a microplate reader at multiple time points to calculate the reaction rate. GSH levels were calculated using a standard curve, normalised to total protein content and expressed as μmol/mg protein.

### 2.10 Cytokine and IgE Measurement

The BALF and conditioned medium were used to measure cytokine levels and IgE levels by enzyme‐linked immunosorbent assay (ELISA). For human samples (conditioned medium from BEAS‐2B cells), the cytokine levels of TNF‐α (Cat# DTA00D, R&D Systems), IL‐1β (Cat# DLB50, R&D Systems), IL‐18 (Cat# DL180, R&D Systems) and IL‐10 (Cat# D1000B, R&D Systems) were measured. For murine samples (BALF), TNF‐α (Cat# MTA00B, R&D Systems), IL‐1β (Cat# MLB00C, R&D Systems), IL‐18 (Cat# DY7625‐05, R&D Systems) and IL‐10 (Cat# M1000B, R&D Systems) were assessed. Mouse IgE levels in BALF were measured using (Cat# 88–50,460‐88, Invitrogen/ThermoFisher). All measurements were performed following the manufacturer's protocol. Briefly, 96‐well plates were coated with capture antibody overnight at 4°C and then blocked with 1% BSA for 1 h at room temperature. Subsequently, samples and standards were added to the plates and incubated for 2 h at room temperature, followed by 2 h of incubation with biotinylated detection antibody. Streptavidin‐horseradish peroxidase (HRP) conjugate was added and incubated for 30 min. After washing the plates with the wash buffer, the substrate solution was added and incubated for 20 min. Following the addition of the stop solution, absorbance was measured using a microplate reader at 450 nm. Cytokine and IgE concentrations were calculated using standard curves.

### 2.11 Animal Studies

Male C57BL/6 mice (8–10 weeks old), purchased from Shanghai Slack Laboratory Animal Co. Ltd., were housed at the animal research centre under standard conditions with a 12:12 h light/dark cycle and ad libitum access to food and water for 1 week prior to experimentation for acclimatisation. To establish the asthma model, mice were initially sensitised with 1% ovalbumin (OVA) administered intraperitoneally, followed by nebulisation with 1% OVA for 14 days, 1 h per day [18, 19]. For gene silencing experiments, mice were transfected with sh‐KIF1B delivered via lentiviral particles (lv‐sh‐KIF1B, Cat# MR215734L3V, OriGene) at a dose of 1 × 10^8 transducing units (TU) per mouse, administered 2 days prior to OVA treatment; lv‐sh‐NC (Cat# TR30021V, OriGene) at the same viral titre served as the negative control. Lentiviral particles were diluted in 50 μL of sterile PBS and administered directly into the lungs via oropharyngeal instillation under light anaesthesia. Eight animals were included in each group, and power analysis calculations (α = 0.05, *β* = 0.20) demonstrated that *n* = 8 per group provides adequate statistical power (80%) to detect meaningful biological differences while minimising animal use according to ethical guidelines.

Following completion of the 2 week OVA treatment protocol, pulmonary function was assessed using whole‐body plethysmography (Buxco Research Systems, USA) 24 h after the final OVA challenge. Mice were anaesthetised and intubated, and airway resistance (cmH2O·s/mL) and lung compliance (mL/cmH2O) were measured under baseline conditions using standardised pressure–volume curves. Then all mice were euthanised using an overdose of ketamine/xylazine, and bronchoalveolar lavage fluid (BALF) was collected through tracheal cannulation using 0.6 mL × 3 aliquots of PBS. Collected BALF was centrifuged at 1000 g for 10 min to separate BALF cells, while supernatants were transferred to separate tubes for subsequent ELISA analysis. BALF cells were resuspended and prepared for cytospin preparation, followed by Diff‐Quik staining (Cat# NC2059480, Fisher Scientific) for differential cell counting. Lung tissues were collected and either snap‐frozen in liquid nitrogen for gene and protein analysis or fixed in 10% formalin for histological staining. The experimental protocol used in this study was approved by the Animal Ethics Committee of The Third People's Hospital of Hubei Province (Approval No. 2023–014). All experimental procedures were performed in accordance with institutional guidelines and national regulations for animal research.

### 2.12 Haematoxylin and Eosin (H&E) Staining

Lung sections (5 μm) prepared from formalin‐fixed paraffin‐embedded (FFPE) blocks were deparaffinised using xylene and rehydrated through a graded ethanol series: 100%, 90%, 75% ethanol (EtOH, Cat# E7023, Sigma‐Aldrich) and distilled water. Subsequently, sections were stained with Harris haematoxylin (Cat# HHS16, Sigma‐Aldrich) for 1 min and differentiated and blued with 0.1% ammonia water (Cat# 320145, Sigma‐Aldrich) for 10 s. Following a brief rinse, sections were differentiated with 95% EtOH for 1 min and counterstained with alcoholic eosin (Cat# HT110116, Sigma‐Aldrich) for 1 min. The slides were then dehydrated through graded EtOH and cleared with xylene before being mounted with mounting medium (Cat# 06522, Sigma‐Aldrich) for light microscopy imaging.

## 3 Statistic Analysis

All experiments were repeated at least three times independently. Data are presented as mean ± standard deviation (SD). Statistical differences among multiple groups were analysed using one‐way analysis of variance (ANOVA) followed by Tukey's post hoc test for multiple comparisons. Comparisons between two groups were performed using Student's t‐test (unpaired, two‐tailed). All statistical analyses were conducted using GraphPad Prism software (version 10.0, GraphPad Software Inc., San Diego, CA, USA). A *p* < 0.05 was considered statistically significant.

## 4 Results

### 4.1 Increased Expression of KIF1B in Asthmatic Responses

**FIGURE 1:**
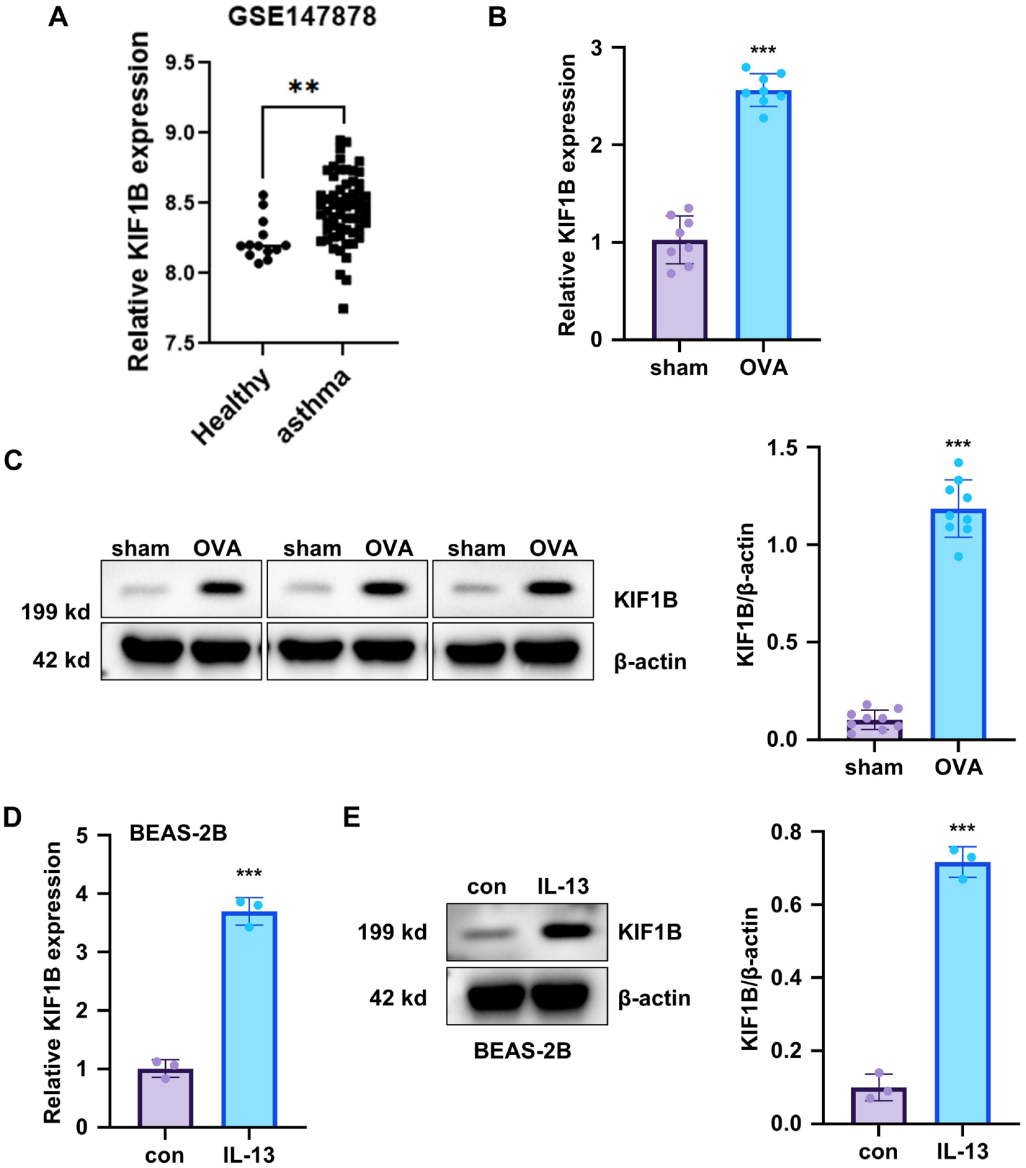
KIF1B expression profiles in different asthmatic models. (A) Gene expression of KIF1B was analysed from the GSE147878 dataset, specifically in bronchial biopsies from asthmatic patients and healthy controls (*n* = 60 for asthmatic patients and *n* = 13 for healthy controls). (B) C57BL/6 mice were treated with 1% ovalbumin (OVA) for 14 days, lung tissues were collected, and KIF1B gene expression was measured by qRT‐PCR (*n* = 8). (C) Protein expression of KIF1B in mouse lung tissues was assessed by Western blot (*n* = 3). (D) BEAS‐2B cells were treated with 10 ng/mL IL‐13 for 24 h, and KIF1B gene expression was measured by qRT‐PCR (*n* = 3). (E) Protein expression of KIF1B in BEAS‐2B cells following IL‐13 treatment was assessed by Western blot (*n* = 3). GAPDH gene was used as an internal reference for qPCR analysis and *β*‐actin was used as the loading control for normalisation in Western blot. Data are presented as mean ± SD (**p < 0.01, ***p < 0.001).

Analysis of the publicly available dataset GSE147878 revealed that KIF1B was significantly overexpressed in bronchial biopsies from asthmatic patients compared to healthy controls (Figure 1A). Consistent with these clinical findings, both mRNA and protein expressions of KIF1B were significantly increased in murine lungs following OVA treatment compared to the control group (Figure 1B,C). To establish an in vitro cell model, we treated BEAS‐2B bronchial epithelial cells with IL‐13, a well‐established asthma‐inducing cytokine [20, 21, 22]. CCK‐8 viability assay revealed a concentration‐dependent decrease in cell viability with an IC50 of approximately 15–20 ng/mL, and we selected 10 ng/mL for subsequent experiments to minimise cytotoxicity while maintaining biological activity (Figure S1). Treatment with IL‐13 resulted in significantly increased mRNA and protein levels of KIF1B compared to untreated controls (Figure 1D,E). Collectively, these results demonstrate that both transcript and protein levels of KIF1B are upregulated across multiple experimental models of asthmatic inflammation, suggesting a potential role for KIF1B in asthma pathogenesis.

### 4.2 Silencing of KIF1B Inhibits Oxidative Stress in IL‐13‐Induced Asthmatic Responses in BEAS‐2B Cells

**FIGURE 2:**
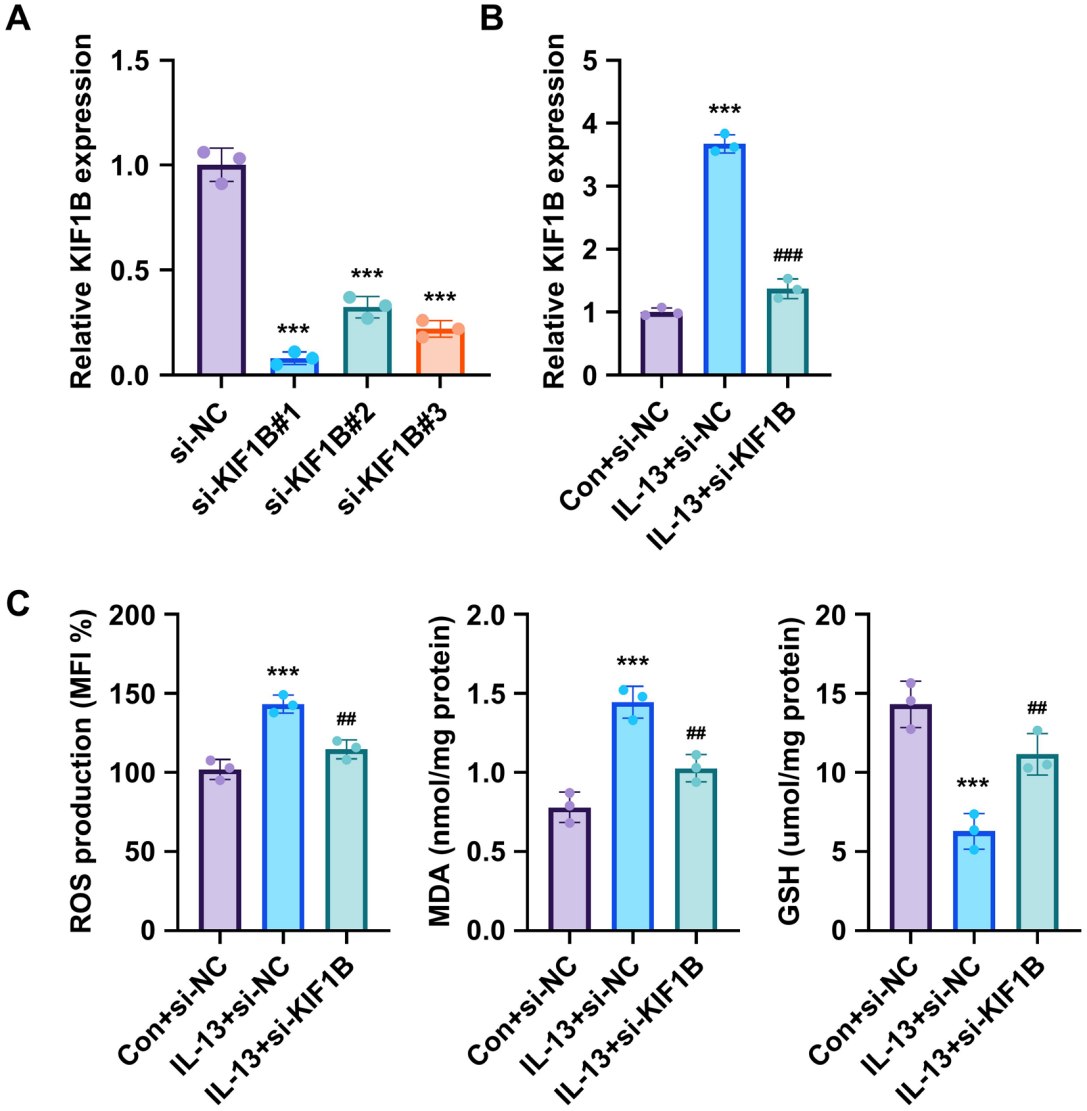
KIF1B silencing inhibits IL‐13‐induced oxidative stress in BEAS‐2B cells. (A) BEAS‐2B cells were transfected with si‐NC (control siRNA), si‐KIF1B#1, si‐KIF1B#2, or si‐KIF1B#3 and KIF1B gene expression was measured by qRT‐PCR. (B) BEAS‐2B cells were transfected with si‐KIF1B and subsequently treated with IL‐13 (10 ng/mL) for 24 h, followed by measurement of KIF1B gene expression by qRT‐PCR. (C) Following si‐KIF1B transfection and IL‐13 treatment, intracellular ROS, MDA and GSH levels were measured using commercial assay kits. The GAPDH gene was used as an internal reference for qPCR analysis. Data are presented as mean ± SD (*n* = 3; ***p < 0.001 vs. control, *p* < 0.01, *p* < 0.001 vs. IL‐13 group).

Following confirmation of increased KIF1B expression across human cohorts, animal and cell models, we further performed a loss‐of‐function investigation of the functional role of KIF1B. Three different siRNAs targeting KIF1B were transfected into BEAS‐2B cells, and si‐KIF1B#1 demonstrated the most effective knockdown of KIF1B gene expression and was therefore selected for subsequent experiments (Figure 2A). KIF1B siRNA transfection successfully suppressed IL‐13‐induced KIF1B upregulation (Figure 2B). IL‐13 treatment of BEAS‐2B cells resulted in significantly decreased GSH levels and increased ROS and MDA levels, indicative of enhanced oxidative stress. Importantly, KIF1B knockdown significantly alleviated these IL‐13‐induced changes in oxidative stress markers (Figure 2C). These findings demonstrate that KIF1B silencing can attenuate IL‐13‐induced oxidative stress in bronchial epithelial cells, suggesting a critical role for KIF1B in mediating oxidative responses in asthmatic inflammation.

### 4.3 KIF1B Knockdown Reduces IL‐13‐Induced Inflammation and Pyroptosis in BEAS‐2B Cells

**FIGURE 3:**
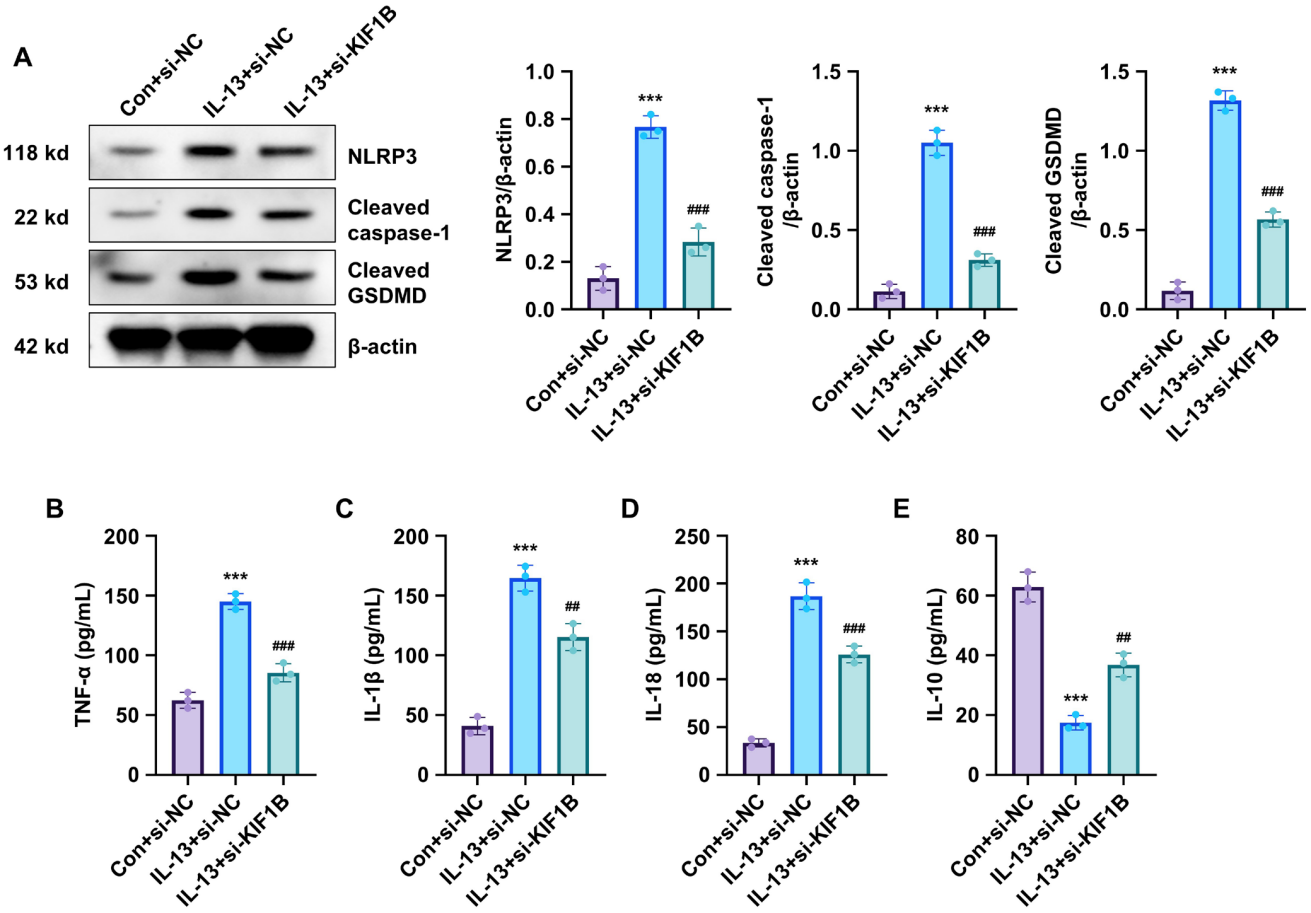
KIF1B inhibition reduces IL‐13‐induced inflammatory responses in BEAS‐2B cells. (A) BEAS‐2B cells were transfected with si‐KIF1B or si‐NC and treated with IL‐13 (10 ng/mL) for 24 h, followed by Western blot analysis of NLRP3, cleaved caspase‐1 and cleaved GSDMD protein expression. Conditioned medium was collected to measure cytokine levels: (B) TNF‐α, (C) IL‐1β, (D) IL‐18 and (E) IL‐10 by ELISA. β‐actin was used as the loading control for normalisation in Western blot. Data are presented as mean ± SD (*n* = 3; ***p < 0.001 vs. control, *p* < 0.01, *p* < 0.001 vs. IL‐13 group).

We next investigated the role of KIF1B in IL‐13‐induced inflammation and pyroptosis. IL‐13 treatment significantly increased the protein expression of key pyroptosis markers, including NLRP3, cleaved caspase‐1 and cleaved GSDMD. Transfection with si‐KIF1B effectively suppressed the overexpression of these pyroptotic proteins (Figure 3A). Similarly, the pyroptosis‐related pro‐inflammatory cytokines released by BEAS‐2B cells, including TNF‐α, IL‐1β and IL‐18, were significantly upregulated following IL‐13 treatment. KIF1B silencing markedly reduced the secretion of these inflammatory mediators (Figure 3B–D). Conversely, the anti‐inflammatory cytokine IL‐10 was significantly decreased by IL‐13 treatment, and this suppression was partially reversed by KIF1B knockdown (Figure 3E). Collectively, these findings demonstrate that KIF1B knockdown can effectively attenuate IL‐13‐induced inflammation and pyroptosis in bronchial epithelial cells.

### 4.4 Overexpression of NLRP3 Reverses the Anti‐Inflammatory Effects of KIF1B Knockdown in BEAS‐2B Cells

**FIGURE 4:**
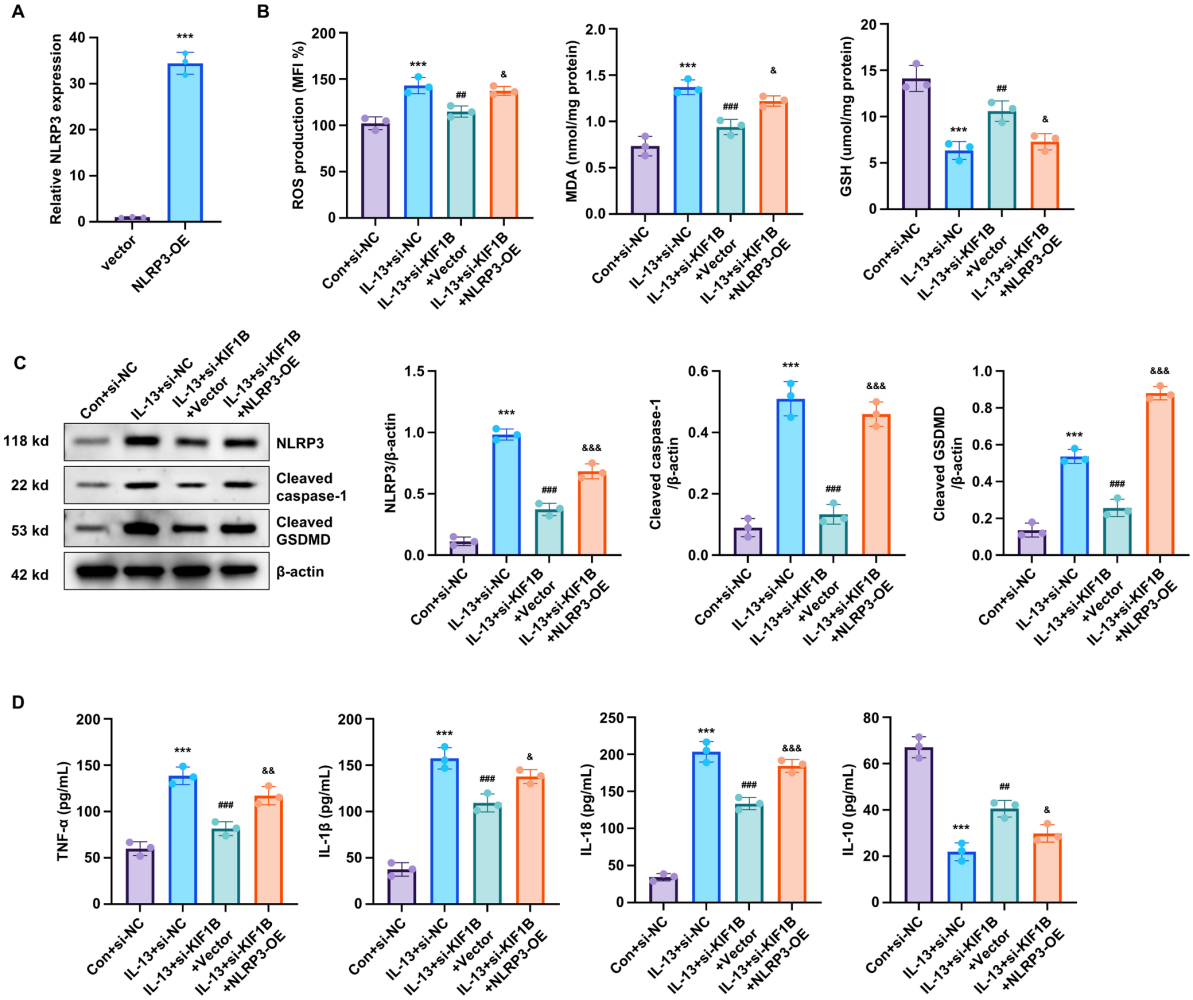
KIF1B regulates IL‐13‐induced inflammation in BEAS‐2B cells via NLRP3. BEAS‐2B cells were transfected with si‐NC, si‐KIF1B + empty vector or si‐KIF1B + NLRP3‐ORF plasmid, with or without IL‐13 treatment. (A) NLRP3 gene expression was measured by qRT‐PCR following NLRP3‐ORF transfection to confirm successful overexpression. (B) Following transfection with si‐KIF1B with or without NLRP3‐ORF and IL‐13 treatment, intracellular ROS, MDA and GSH levels were measured. (C) Protein expression of NLRP3, cleaved caspase‐1 and cleaved GSDMD was assessed by Western blot. (D) Cytokine levels (TNF‐α, IL‐1β, IL‐18 and IL‐10) in conditioned medium were measured by ELISA. GAPDH gene was used as an internal reference for qPCR analysis, and β‐actin was used as the loading control for normalisation in Western blot. Data are presented as mean ± SD (*n* = 3; ***p < 0.001 vs. control, *p* < 0.01, *p* < 0.001 vs. IL‐13 group, & *p* < 0.05, & *p* < 0.01 vs. IL‐13 + si‐KIF1B group).

To elucidate the mechanism underlying KIF1B's anti‐inflammatory effects, we performed NLRP3 overexpression experiments. Transfection with the NLRP3‐ORF plasmid successfully elevated NLRP3 expression levels (Figure 4A). NLRP3 overexpression reversed the protective effects of KIF1B knockdown on oxidative stress, re‐elevating ROS and MDA levels while decreasing GSH levels (Figure 4B). Similarly, NLRP3 overexpression restored the protein expressions of NLRP3, cleaved caspase‐1 and cleaved GSDMD that were suppressed by KIF1B silencing (Figure 4C). The normalised cytokine profile achieved by KIF1B knockdown—reduced pro‐inflammatory cytokines (TNF‐α, IL‐1β, IL‐18) and restored IL‐10—was also reversed by NLRP3 overexpression (Figure 4D). These findings demonstrate that KIF1B regulates IL‐13‐induced inflammation and pyroptosis through the NLRP3 inflammasome pathway.

### 4.5 Silencing of KIF1B Alleviates OVA‐Induced Lung Injury in Mice

**FIGURE 5:**
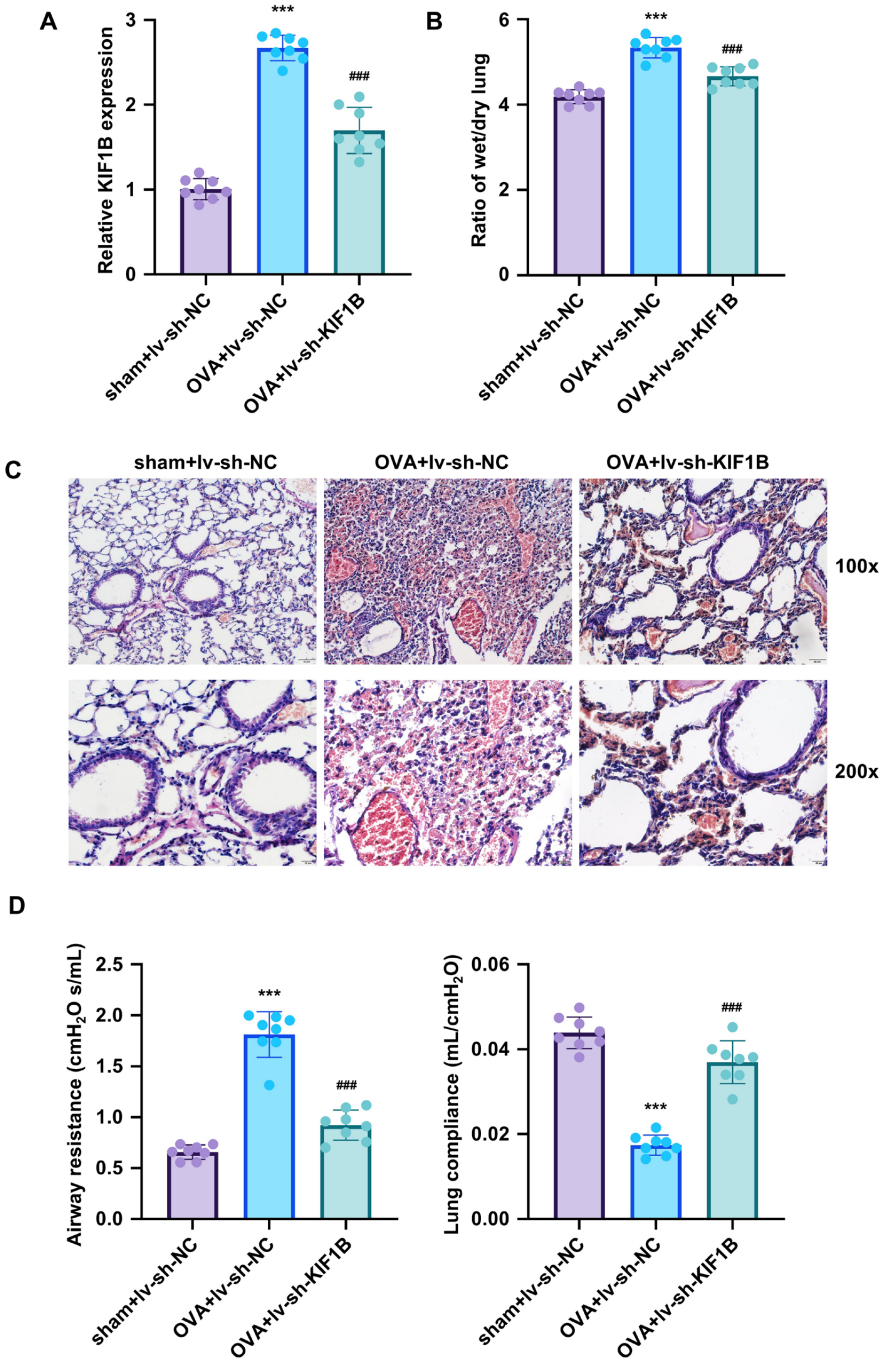
KIF1B knockdown protects against OVA‐induced lung injury in mice. C57BL/6 mice were infected with lentiviral particles carrying sh‐KIF1B or sh‐NC, and subsequently treated with OVA for 14 days to induce asthmatic responses. Lung tissues were collected at sacrifice. (A) KIF1B gene expression in lung homogenates from control, OVA‐treated and OVA + sh‐KIF1B groups was measured by qRT‐PCR. (B) Lung wet/dry (W/D) weight ratios were calculated to assess pulmonary oedema. (C) Formalin‐fixed paraffin‐embedded (FFPE) lung sections were stained with H&E to evaluate histopathological changes. (D) Pulmonary function assessment in the OVA‐induced asthma model. Airway resistance (left panel) and lung compliance (right panel) were measured by plethysmography. The GAPDH gene was used as an internal reference for qPCR analysis. Data are presented as mean ± SD (*n* = 8; ***p < 0.001 vs. control, *p* < 0.001 vs. OVA group).

To validate the protective effects of KIF1B silencing in vivo, C57BL/6 mice were treated with sh‐KIF1B lentiviral particles followed by OVA challenge for 14 days to induce asthmatic responses. The administration of sh‐KIF1B lentiviral particles significantly decreased KIF1B gene expression, while OVA treatment led to significant upregulation compared to the control group (Figure 5A). OVA treatment increased the lung tissue wet/dry (W/D) ratio, indicative of pulmonary oedema, which was significantly reduced by KIF1B knockdown (Figure 5B). Furthermore, histopathological analysis of H&E‐stained lung sections revealed that KIF1B silencing markedly attenuated OVA‐induced lung injury, including reduced inflammatory cell infiltration and tissue damage (Figure 5C). Pulmonary function analysis revealed that OVA‐induced asthma significantly increased airway resistance and decreased lung compliance compared to controls, while KIF1B knockdown effectively restored both parameters towards normal values (Figure 5D). These findings demonstrate that KIF1B knockdown provides protective effects against OVA‐induced lung injury.

### 4.6 KIF1B Knockdown Attenuates OVA‐Induced Oxidative Stress in Pulmonary Tissues

**FIGURE 6:**
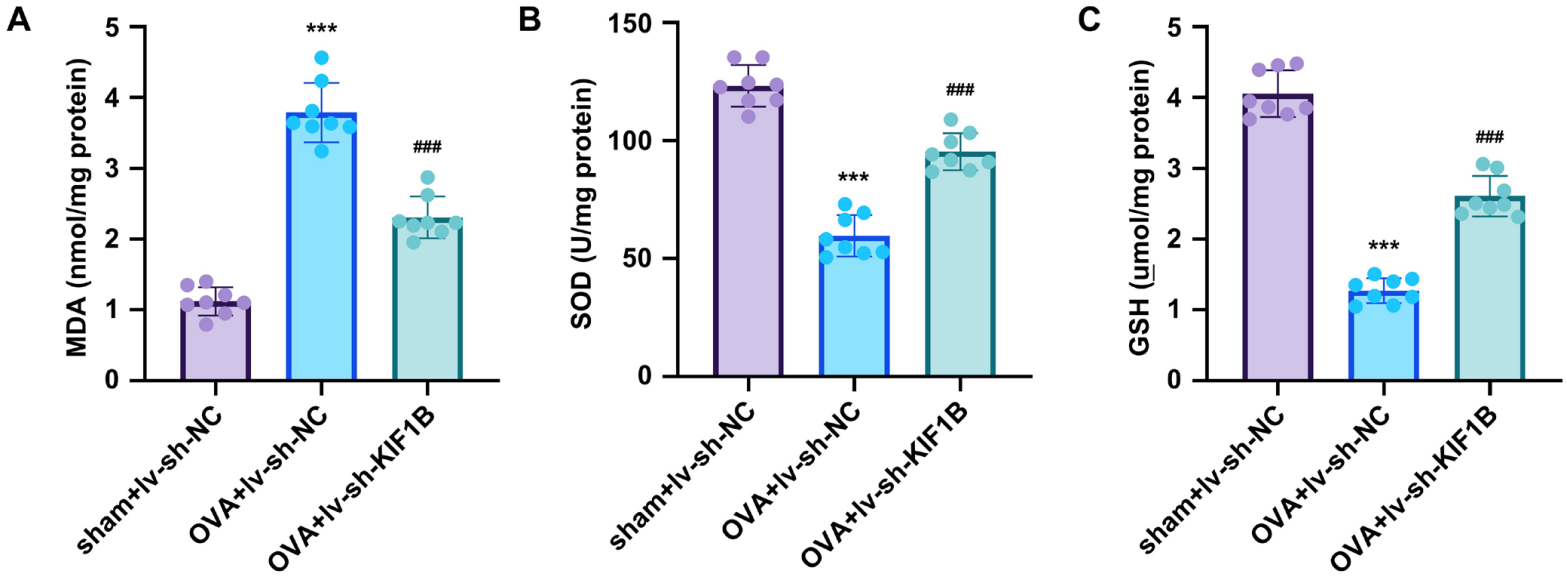
KIF1B inhibition reduces oxidative stress in asthmatic responses. C57BL/6 mice were infected with lentiviral particles carrying sh‐KIF1B or sh‐NC, and subsequently treated with OVA for 14 days to induce asthmatic responses. Lung homogenates were analysed for oxidative stress markers: (A) MDA, (B) SOD and (C) GSH levels. Data are presented as mean ± SD (*n* = 8; ***p < 0.001 vs. control, *p* < 0.001 vs. OVA group).

Following the demonstration that KIF1B knockdown significantly protected against OVA‐induced lung injury, we examined oxidative stress markers to understand KIF1B's role in regulating oxidative responses in asthmatic progression. OVA treatment significantly increased the levels of oxidative stress markers MDA and SOD in lung tissues, both of which were effectively reduced by KIF1B knockdown (Figure 6A,B). Conversely, GSH levels, an important antioxidant marker, were significantly decreased following OVA treatment. KIF1B silencing substantially restored GSH levels (Figure 6C). These findings demonstrate that KIF1B knockdown can alleviate OVA‐induced oxidative stress in the OVA‐induced asthma model.

### 4.7 Silencing KIF1B Attenuates OVA‐Induced Inflammation and Pyroptosis in Pulmonary Tissues

**FIGURE 7:**
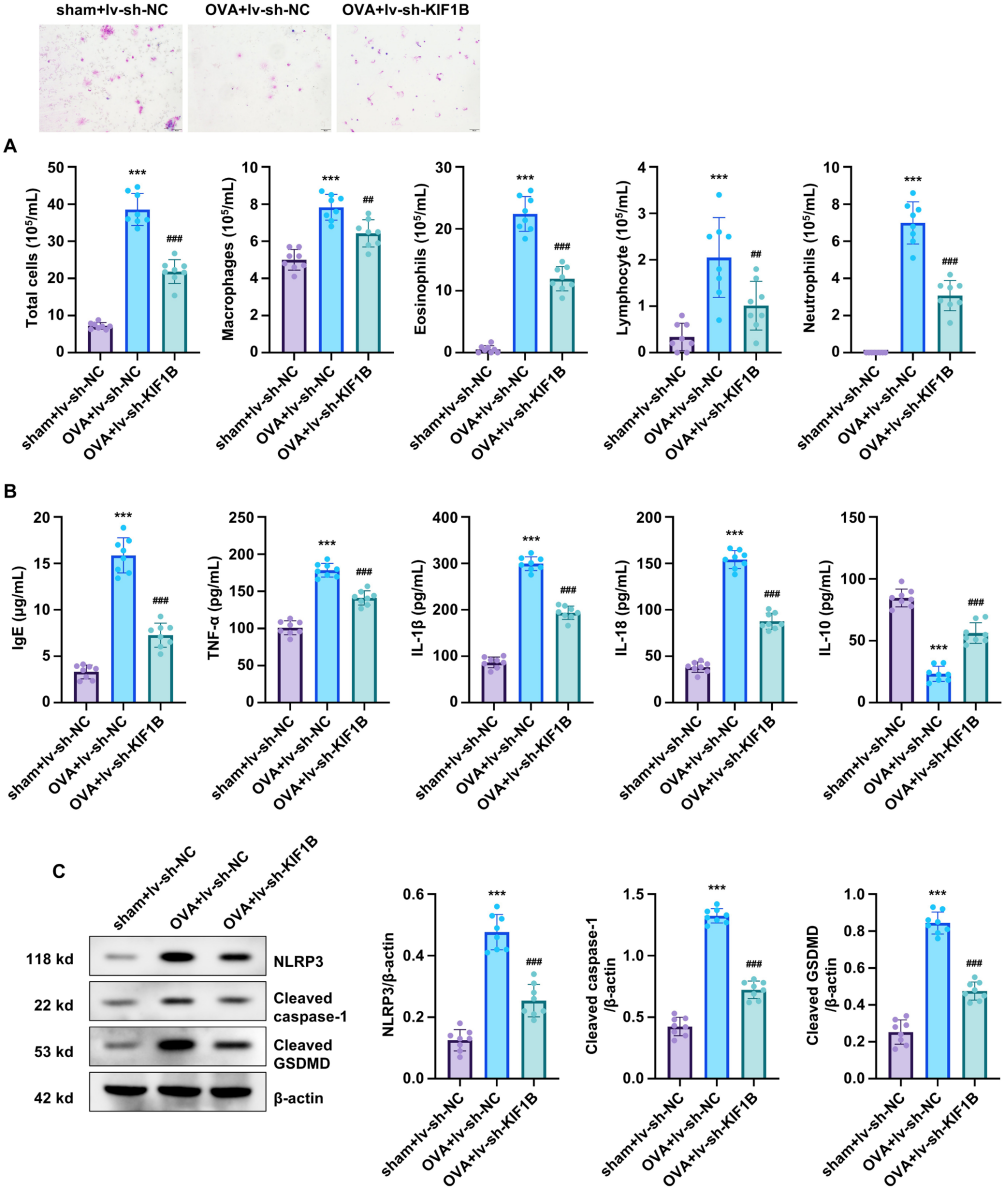
KIF1B knockdown reduces OVA‐induced inflammation and pyroptosis in mouse pulmonary tissues. C57BL/6 mice were infected with lentiviral particles carrying sh‐KIF1B or sh‐NC, and subsequently treated with OVA for 14 days to induce asthmatic responses. Lung tissues were collected at sacrifice. (A) Wright‐Giemsa staining was used to quantify total cells, macrophages, eosinophils, lymphocytes and neutrophils in BALF. (B) IgE, TNF‐α, IL‐1β, IL‐18 and IL‐10 levels in BALF were measured by ELISA. (C) Protein levels of NLRP3, cleaved caspase‐1 and cleaved GSDMD in lung homogenates were assessed by Western blot. β‐actin was used as the loading control for normalisation in Western blot. Data are presented as mean ± SD (*n* = 8; ***p < 0.001 vs. control, *p* < 0.01, *p* < 0.001 vs. OVA group).

We further examined inflammatory responses and pyroptosis in pulmonary tissues. OVA treatment significantly increased total cell and immune cell counts, including macrophages, eosinophils, lymphocytes and neutrophils in bronchoalveolar lavage fluid (BALF), all of which were markedly reduced by KIF1B silencing (Figure 7A). Similarly, allergic immunoglobulin IgE levels and pro‐inflammatory cytokines (TNF‐α, IL‐1β and IL‐18) were significantly upregulated in the OVA group and substantially decreased in the OVA+sh‐KIF1B group (Figure 7B). Conversely, the anti‐inflammatory cytokine IL‐10 was downregulated following OVA treatment and was restored by KIF1B knockdown (Figure 7B). The pyroptosis‐related proteins NLRP3, cleaved caspase‐1 and cleaved GSDMD in pulmonary tissues were increased after OVA treatment and significantly reduced in the OVA+sh‐KIF1B group compared to the OVA group (Figure 7C). These findings demonstrate that KIF1B knockdown effectively protects against OVA‐induced pulmonary inflammation and pyroptosis.

## 5 Discussion

This study investigated the role of KIF1B in asthma, particularly focusing on the mechanisms underlying inflammatory responses and pyroptosis regulation, utilising both in vitro and in vivo experimental models. The findings reveal that KIF1B plays a critical role in modulating inflammatory processes in asthmatic progression. Asthma, characterised as a chronic inflammatory airway disease, typically presents with airway hyperresponsiveness, excessive mucus production and airway obstruction. In severe cases, asthmatic responses can be life‐threatening upon exposure to common environmental allergens such as pollen and house dust mites [23, 24]. Despite significant therapeutic advances, no curative treatment currently exists to completely resolve asthma. Comprehensive mechanistic studies are essential to elucidate asthma pathogenesis and develop more effective therapeutic interventions [25]. This investigation demonstrates that KIF1B inhibition represents a promising therapeutic approach for attenuating asthma development through the suppression of inflammatory progression.

Our data demonstrated that KIF1B expression was significantly upregulated in human asthmatic patients and in both in vivo and in vitro models, suggesting that KIF1B plays an essential role in regulating asthmatic responses. This finding is further supported by our demonstration that silencing KIF1B in either mouse lungs or BEAS‐2B cells effectively attenuated OVA/IL‐13‐induced oxidative stress, inflammation and lung injury. KIF1B was originally discovered as a mitochondrial transporter [26] and has been extensively studied for its role in regulating neuronal inflammation and apoptosis, while limited studies have focused on its pulmonary functions [27, 28]. Consistent with our findings of KIF1B's inflammatory regulatory role, a previous study demonstrated that KIF5B, a kinesin family member closely related to KIF1B, regulates epithelial–mesenchymal transition through the PI3K/AKT/mTOR inflammatory pathway in endometrial cancer [29]. Our results extend these findings by demonstrating that KIF1B is essential in regulating NLRP3 inflammasome activation in asthmatic progression, as evidenced by our rescue experiments showing that NLRP3 overexpression reversed the protective effects of KIF1B silencing. These findings suggest that inhibitors targeting KIF1B could represent a potential therapeutic intervention for asthma prevention and treatment.

Moreover, the results from this study also indicated that KIF1B regulates asthma‐associated pyroptosis, defined as a form of programmed cell death characterised by inflammatory responses [30]. KIF1B knockdown significantly reduced the expression of key pyroptotic proteins, including NLRP3, cleaved caspase‐1 and cleaved GSDMD, which are established markers for pyroptosis [31], in both lung tissues and cellular models. Concurrently, the pro‐inflammatory cytokines TNF‐α, IL‐1β and IL‐18, which serve as established pyroptotic markers [32], were elevated in asthmatic responses but significantly decreased following KIF1B knockdown, as demonstrated in our ELISA analyses. These findings suggest that KIF1B promotes inflammation and pyroptosis in asthmatic progression, likely through activation of the NLRP3/cleaved caspase‐1/GSDMD inflammasome pathway [33]. The inverse regulation of IL‐10 levels observed in our study further confirmed that pyroptosis was effectively inhibited and asthma‐associated lung injury was protected upon KIF1B silencing [33, 34, 35]. Importantly, our mechanistic studies using NLRP3 overexpression validated this pathway, as NLRP3 restoration reversed the protective effects of KIF1B knockdown on both oxidative stress and inflammatory markers.

While we have demonstrated that KIF1B represents a promising novel therapeutic target for asthma treatment through its effective prevention of oxidative stress and inflammation‐mediated pyroptosis/lung injury, the study has several limitations that warrant consideration. First, the OVA‐induced asthma model, while widely used, has limited translational relevance as OVA does not naturally induce allergic responses in humans [36]. Future studies should incorporate more clinically relevant allergens, such as house dust mites (HDM), which better recapitulate human asthmatic pathophysiology [23]. Second, asthma pathogenesis involves multiple complex mechanisms beyond inflammatory responses that require further investigation, including epithelial barrier dysfunction, altered airway resistance, abnormal tissue repair processes and airway remodelling [23, 37, 38, 39, 40]. Third, our study primarily focused on the acute inflammatory phase, while chronic asthma involves sustained airway remodelling and structural changes that may be differentially regulated by KIF1B. Understanding how KIF1B modulates these diverse histological and functional changes throughout different stages of asthmatic progression will be crucial for determining optimal intervention strategies. Additionally, dose–response studies and long‐term safety evaluations of KIF1B inhibition will be necessary before clinical translation. Furthermore, the mechanism by which KIF1B regulates NLRP3 inflammasome activation remains unknown and warrants further investigation.

In conclusion, this study elucidates the critical role of KIF1B in asthma pathogenesis and establishes its potential as a novel therapeutic target. Our findings demonstrate that KIF1B inhibition effectively attenuates oxidative stress, inflammation and pyroptosis through modulation of the NLRP3 inflammasome pathway in asthmatic progression. These mechanistic insights provide evidence supporting the development of KIF1B‐targeted therapies as innovative therapeutic interventions for asthma management. Future clinical investigations focusing on KIF1B inhibitors may offer new avenues for improving patient outcomes in asthmatic diseases.

## Author Contributions

C.Q. Jiang and J. Li mainly participated in literature search, study design, writing and critical revision. Y. Gao and JC Wang mainly participated in data collection, data analysis and data interpretation, and in preparing Figures 1–7. All authors read and approved the final manuscript.

## Funding

The authors have nothing to report.

## Ethics Statement

This study was approved by the Animal Ethics Committee of The Third People's Hospital of Hubei Province (Approval No. 2023–014). The animal experiment was carried out in compliance with the ARRIVE guidelines (https://arriveguidelines.org).

This study did not involve direct patient recruitment. All human data used were obtained from publicly available sources that have obtained prior ethical approval and informed consent.

## Consent

The authors have nothing to report.

## Conflicts of Interest

The authors declare no conflicts of interest.

## Supporting information


**Figure S1:** IL‐13 dose–response curve and cytotoxicity assessment in BEAS‐2B cells.
BEAS‐2B cells were treated with serial two‐fold dilutions of IL‐13 (0.78125–400 ng/mL) for 24 h, and cell viability was assessed using CCK‐8 assay. The IC50 value was calculated to be approximately 15–20 ng/mL. Data are presented as mean ± SD (*n* = 3).

## Data Availability

The data that support the findings of this study are available from the corresponding author upon reasonable request.
