# Supplementary figures and images for: KIF1B Regulates NLRP3‐Mediated Pyroptosis in Asthma Progression
Source: J Cell Mol Med. 2025 Dec 12;29(23):e70975. doi: 10.1111/jcmm.70975 (PMC12699257; doi:10.1111/jcmm.70975)

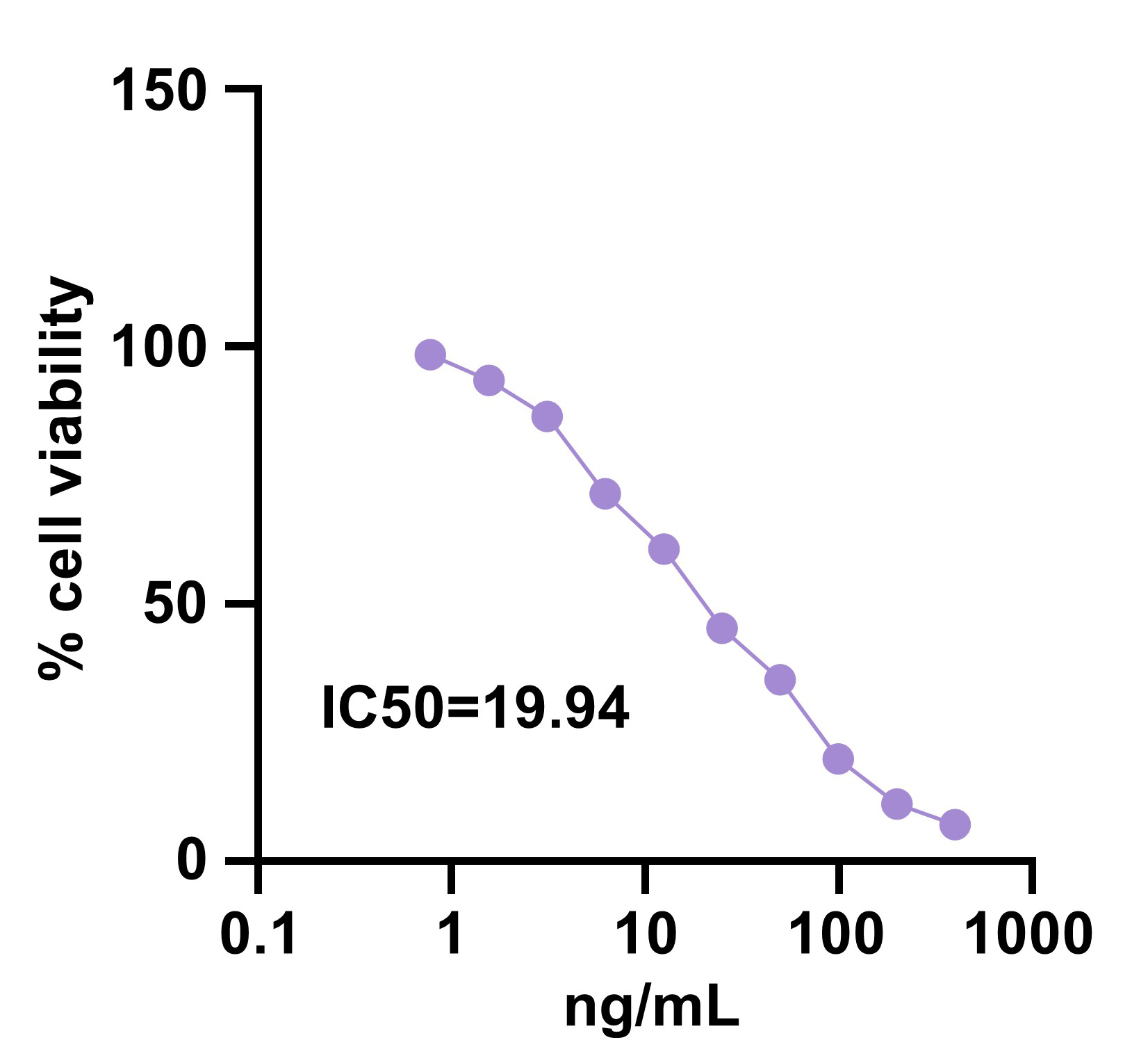

Supplement: Supplementary file 1 — Figure S1: IL‐13 dose–response curve and cytotoxicity assessment in BEAS‐2B cells. BEAS‐2B cells were treated with serial two‐fold dilutions of IL‐13 (0.78125–400 ng/mL) for 24 h, and cell viability was assessed using CCK‐8 assay. The IC50 value was calculated to be approximately 15–20 ng/mL. Data are presented as mean ± SD (n = 3). [file JCMM-29-e70975-s001.jpg]
